# The Impact of Probiotics, Prebiotics, and Synbiotics during Pregnancy or Lactation on the Intestinal Microbiota of Children Born by Cesarean Section: A Systematic Review

**DOI:** 10.3390/nu14020341

**Published:** 2022-01-14

**Authors:** Sandra Martín-Peláez, Naomi Cano-Ibáñez, Miguel Pinto-Gallardo, Carmen Amezcua-Prieto

**Affiliations:** 1 Department of Preventive Medicine and Public Health, Faculty of Medicine, Universidad de Granada, 18016 Granada, Spain; ncaiba@ugr.es (N.C.-I.); miguelpingal@correo.ugr.es (M.P.-G.); carmezcua@ugr.es (C.A.-P.); 2 Instituto de Investigación Biosanitaria de Granada, 18012 Granada, Spain; 3 Consortium for Biomedical Research in Epidemiology and Public Health (CIBERESP), 28029 Madrid, Spain

**Keywords:** probiotics, prebiotics, synbiotics, gut microbiota, pregnancy, cesarean section

## Abstract

The gut microbiota is a key factor in the correct development of the gastrointestinal immune system. Studies have found differences between the gut microbiota of newborns delivered by cesarean section compared to those vaginally delivered. Our objective was to evaluate the effect of ingestion of probiotics, prebiotics, or synbiotics during pregnancy and/or lactation on the development of the gut microbiota of the C-section newborns. We selected experimental studies in online databases from their inception to October 2021. Of the 83 records screened, 12 met the inclusion criteria. The probiotics used belonged to the genera *Lactobacillus*, *Bifidobacterium*, *Propionibacterium*, and *Streptococcus*, or a combination of those, with dosages varying between 2 × 10^6^ and 9 × 10^11^ CFU per day, and were consumed during pregnancy and/or lactation. Probiotic strains were combined with galacto-oligosaccharides, fructo-oligosaccharides, or bovine milk-derived oligosaccharides in the synbiotic formulas. Probiotic, prebiotic, and synbiotic interventions led to beneficial gut microbiota in cesarean-delivered newborns, closer to that in vaginally delivered newborns, especially regarding *Bifidobacterium* colonization. This effect was more evident in breastfed infants. The studies indicate that this beneficial effect is achieved when the interventions begin soon after birth, especially the restoration of bifidobacterial population. Changes in the infant microbial ecosystem due to the interventions seem to continue after the end of the intervention in most of the studies. More interventional studies are needed to elucidate the optimal synbiotic combinations and the most effective strains and doses for achieving the optimal gut microbiota colonization of C-section newborns.

## 1. Introduction

The gut microbiota (GM) is the set of microorganisms coexisting in the gut of an individual [1]. It plays an active role in the development and maturation of the gastrointestinal mucosal immune system (GALT) and in the defense against intestinal pathogens [1,2]. At birth, the newborn presents an immature immune system, which requires immunogenic stimuli from the developing GM for proper maturation [1,2]. It seems that the development of the GM begins in the womb, contrary to the commonly accepted paradigm of the fetus as a sterile organism [3,4]. In fact, species from the genera *Staphylococcus*, *Lactobacillus*, and *Bifidobacterium*, have been identified in the meconium, placenta, and amniotic fluid of neonates of healthy pregnant women [3,4]. This early GM can be affected by external factors such as the route of birth and diet [2], and is essential for infant and adult health [5,6].

Differences in the degree of gut microbiota development between children born by C-section and those born by vaginal delivery have been found, with the former having a less developed microbiota [7,8,9]. The gut microbiota of C-section newborns contains lower numbers of species of the genera *Bifidobacterium*, *Streptococcus*, and *Lactobacillus*, and higher numbers of potentially pathogenic bacteria, such as *Clostridium perfringens* or *Escherichia coli* [10,11,12], compared to vaginally delivered newborns. Although a commonly given explanation is that during passage through the vaginal canal, the newborn acquires different beneficial bacteria that colonize the intestinal tract [7,9], the lack of exposure to vaginal microbiota is unlikely to be the sole contributing factor. Other elements such as intrapartum antibiotic administration, differences in feeding behaviors, maternal obesity, gestational age, limited early skin-to-skin contact after birth, or absence of labor [13,14,15] could also be important drivers of the C-section newborn’s GM. Amongst them, neonate feeding seems to play a very important role. Researchers have shown that women who deliver by C-section are less likely to breastfeed, or will delay breastfeeding initiation [16,17]. This is of great importance for GM colonization, since breast milk contains a plethora of beneficial bacteria essential for the optimum immune development and the intestinal colonization in the newborn [18]. The bacterial composition of breast milk is closely related to that of the GM of babies, indicating the existence of the passage of bacteria from mother to the child during the lactation process [2]. Therefore, the lactation process seems to be a key factor in the development and correct establishment of the GM in children born vaginally and by cesarean section [19,20]. In this sense, it has been suggested that external supplementation with probiotics (live microorganisms that, after ingestion in specific numbers, confer health benefits to the host [21]), prebiotics (a substrate that is selectively utilized by host microorganisms, conferring a health benefit [22]), or synbiotics (a mixture comprising live microorganisms and substrate(s) used selectively by host microorganisms that confers health benefits to the host [23]) in the mother could positively contribute to the colonization of the GM of the newborn and therefore contribute to a good immune development in a natural way [24], which is especially important for C-section newborns. We aimed to evaluate, in published analytical experimental studies, the effect of the probiotic, prebiotic, or synbiotic ingestion during pregnancy and/or lactation on the colonization of the GM of C-section newborns.

## 2. Materials and Methods

This systematic review was carried out following prospective registration (PROSPERO ID: CRD42021241641) and reported according to the PRISMA statement [25].

### 2.1. Literature Search

Searches were conducted in the PubMed, Web of Science, and Scopus databases from their inception to October 2021. ScienceDirect was used as additional source. Keywords used were probiotic, synbiotic, prebiotic, *Lactobacillus*, *Bifidobacterium,* oligosaccharides, pregnancy, lactation, breastfeeding, and cesarean. Boolean descriptors AND, OR, and NOT were used, as well as performing truncations (*) of the different terms. To further define the results, additional filters were used. The searching strings used in each of the databases are presented in Table S1.

### 2.2. Selection Criteria

We included experimental studies published from the time of inception, until October 2021, written in English with full text available, and conducted in humans. Studies that did not refer to the efficacy of the use of probiotics, prebiotics, or synbiotics during pregnancy or lactation on the gut microbiota of C-section newborns, and studies with unclear data information about the interventions were excluded. The selection process is further described in Section 3.1.

### 2.3. Data Extraction and Analysis

Data were extracted by CA-P and NC-I, and contrasted by SM-P. From each selected publication, information on authorship, year of publication, place of completion of the study, study population, microorganisms and/or oligosaccharides and dosage used, administration vehicle, intervention duration, and outcomes regarding gut microbiota of neonates was obtained.

### 2.4. Quality Assessment

Selected studies were evaluated by C.A.-P. and N.C.-I., and disagreements were contrasted by S.M.-P. using the 2020 update of the Cochrane Risk of Bias (RoB2) assessment tool [26]. Five areas were evaluated in risk of bias: selection, performance, detection, attrition, and reporting. Every single item was evaluated to have a high, low, or unclear risk of bias and an overall estimation was obtained for each study, which was classified as a low, medium, or high risk of bias.

## 3. Results

### 3.1. Selection Process

**Figure 1:**
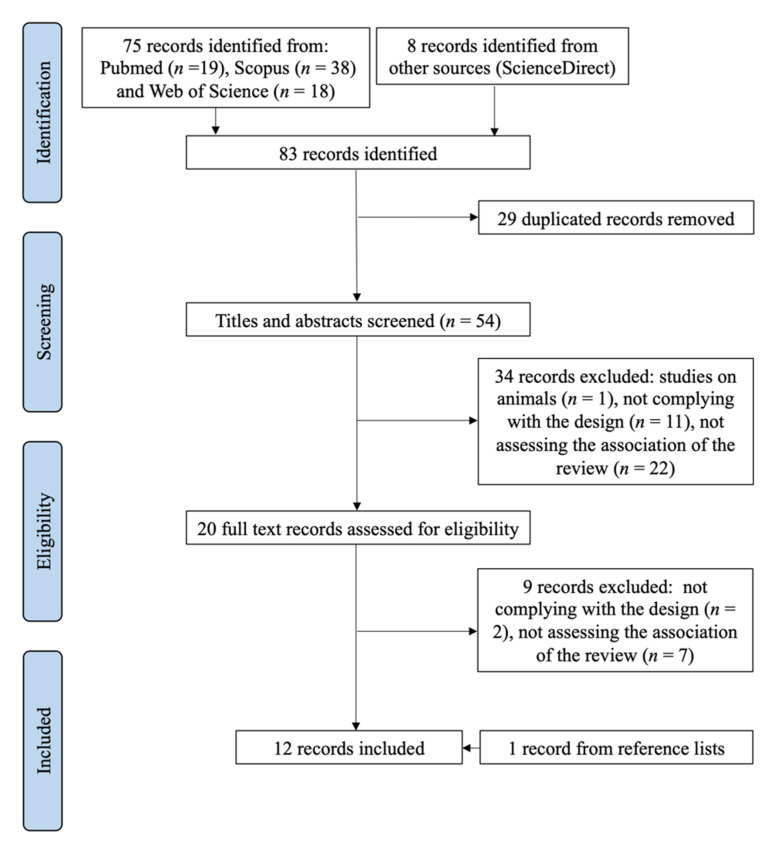
Flow diagram (PRISMA). Systematic selection of studies for review.

The selection process is presented in Figure 1, according to the PRISMA flow diagram [27].

The electronic search using the strategy previously described yielded a total of 83 records (19 PubMed, 38 Scopus, 18 Web of Science, 8 ScienceDirect). After duplicates were deleted, 54 titles and abstracts were screened. Of these, 34 articles not meeting the eligibility criteria (1 study on animals, 11 not complying with the design, 22 not assessing the association of the review) were dismissed. From the 20 articles that were fully read, 9 did not meet the eligibility criteria (2 not complying with the design, 7 not assessing the association of the review) and were also dismissed. The reference lists of the remaining 11 articles revealed 1 further citation. Finally, 12 records were included in the systematic review.

### 3.2. Characteristics of Studies Selected

Information on authorship and year of publication, study population, type and duration of the intervention, and outcomes regarding gut microbiota are shown in Table 1.

The studies included in this review were published between 2013 and 2021. Most of them were conducted in Asia (Thailand [28,34,39], Indonesia [28], Singapore [34], and the Philippines [38]), followed by Europe (Finland [36], Greece [30,32], Italy [39], Germany [33], and Poland [37]), South Africa [31], and the USA [35].

Most of the studies were double-blind, randomized, controlled trials [29,30,31,32,33,34,36,38,39], two were randomized controlled trials [35,37], and one was not randomized [28].

From the twelve studies included, two investigated the effect of the interventions in the mother and the offspring [29,36], whereas the remaining ten studies investigated the effect of the interventions only in the infants. Three studies used only C-section newborns as the study population [30,37,39]; the remaining studies investigated the effect of the interventions in infants born by both delivery types (vaginally and C-section). The sample size varied from 40 [32] to 422 [36].

Most of the studies used probiotics as unique intervention [28,29,30,32,33,35,37], followed by synbiotics [31,39] and prebiotics [38]. In addition, one study investigated combinations of either probiotics and synbiotics [36] or prebiotics and synbiotics [34]. Interventions were compared with infant formula [31,32,33,34,38,39], breastfeeding [30,35], mixed feeding [28,37], corn starch [29], or microcrystalline cellulose [36] as their control groups. Three studies included a breastfeeding reference group in addition to the control group [33,38,39].

When the intervention included the pregnant female, this took place from week 36 of gestation until delivery [29,36]. In infants, most of the interventions started at birth [28,29,30,31,33,36,37,39] or within 3 days after delivery [32,34]. The interventions in the remaining studies started later: one week after birth [35] or at almost one month of life [38].

All the studies found beneficial effects of the interventions on the gut microbiota of CD infants.

### 3.3. Interventions with Probiotics

All the selected studies reported information about the specific strains used. Studies investigating probiotics used either a single strain, belonging either to the genera *Bifidobacterium* [28,30,35] or *Lactobacillus* [32], or multi-strain combinations [29,33,36,37]. Some of the multi-strain combinations included, in addition to *Bifidobacterium* or *Lactobacillus* strains, others belonging to the genera *Streptococcus* [29] or *Propionibacterium* [36].

Regarding the genus *Bifidobacterium*, the most commonly used strains in probiotic interventions belonged to the species *Bifidobacterium breve* [29,33,36,37,39], *Bifidobacterium longum* [29,33,35], and *Bifidobacterium animalis* [28,30]. In contrast, in the interventions with probiotics using strains belonging to the genus *Lactobacillus*, the variety of species was higher (*L. acidophilus*, *L. delbrueckii subsp. bulgaricus*, *L. GG*, *L. paracasei*, *L. plantarum*, *L. reuteri*, *L. rhamnosus*) [29,32,36,37].

The doses used were expressed in CFU per gram [30,31,33], CFU per mL or per liter [32,34,39], or CFU per day [29,35,36,37].

### 3.4. Interventions with Prebiotics

From the twelve included studies, only one used a prebiotic as unique intervention [38]. In another study, the intervention with prebiotics was compared with an intervention with synbiotics [34]. The prebiotics used were milk-derived oligosaccharides [38] and a combination of short-chain galacto-oligosaccharides and long-chain fructo-oligosaccharides [34].

### 3.5. Interventions with Synbiotics

Four studies used synbiotics as intervention [31,34,36,39], with newborns as the population under study. Strains of *Bifidobacterium breve* were the most used in the synbiotic combinations [34,36,39]; in one study together with other strains from the genus *Lactobacillus* in a multi-strain mixture [36]. The prebiotic components were either galacto-oligosaccharides alone [36] or in combination with fructo-oligosaccharides [34,39], and bovine milk-derived oligosaccharides [31].

### 3.6. Stool Sample Collection and Microbial Analysis Methods

Fecal sample collection was performed at home in all the studies. In all the studies where this process was described in detail, the sample was stored either in domestic freezers [35,37] or in fridges [32,33,34,39] at home prior to delivery to the place of analysis, either as collected [32,34,35,39], mixed with storage media [30,37], or maintained in anaerobic conditions [33]. At the place of analysis, samples were stored at −40 °C [32], −70 °C [37], or −80 °C [29,30,33,34,35,39] until subsequent analysis. None of the studies specified a maximum storage time required for analysis.

All the studies used a variety of 16S RNA-based methods for the analysis of the IM; some of them were combined with traditional culture methods [30,31,37].

### 3.7. Study Quality Assessment

Table S2 shows the evaluation of the methodological quality of the 12 studies included in this systematic review. Two studies were considered to have high overall risk of bias [28,35], three had an unclear overall risk of bias [31,34,39], and seven had a low overall risk of bias [29,30,32,36,37,38].

## 4. Discussion

The differences found in the gut microbiota of vaginally delivered newborns and C-section newborns show a more immature and less effective GM in the C-section newborns. This causes susceptibility to develop certain metabolic or immune disorders [7,9]. Probiotic, prebiotic, and synbiotic interventions led to a beneficial gut microbiota in C-section newborns, closer to that of vaginally delivered newborns, especially regarding *Bifidobacterium* colonization.

Regarding probiotic interventions, the effects observed on GM were more evident when multi-strain combinations were used [32,33]. In synbiotic formulas, probiotic strains (all from *Bifidobacterium* genus) were combined with galacto-oligosaccharides, fructo-oligosaccharides, or bovine milk-derived oligosaccharides. This could represent an optimal strategy to achieve the restoration of GM in CD-delivered infants. It is thought that interventions with *Bifidobacterium* strains alone could be insufficient to promote an effect on GM [40]. This could be due to the difficulty of achieving permanent colonization of the infant gut due to competition with autochthonous microbiota. In this sense, it has been suggested that the combination of *Bifidobacterium* with a prebiotic or with breastfeeding, which provides milk oligosaccharides, would be more successful regarding colonization [41]. In fact, Chua et al. [34] found that a synbiotic intervention combining *B. breve* M16V with galacto- and fructo-oligosaccharides increased infant gut colonization by the probiotic strain and by other members of the *Bifidobacterium* genus compared to formula-fed infants [34]. In C-section infants, this colonization was similar to that one of vaginally delivered infants. In addition, Cooper et al. [31] found a strong bifidogenic effect of a synbiotic preparation containing *B. lactis* CNCM I-3446 and bovine milk-derived oligosaccharides, which was more evident in C-section newborns. This is of a great importance due to the relevance of *Bifidobacterium* colonization in early life immune programming [42,43].

The studies indicate that the sooner the intervention begins, the more successful the effect achieved [41], since the first three months of life are a key window for GM recovery in C-section infants, especially regarding *Bifidobacterium*. In fact, most of the studies included in this review started the interventions immediately after birth, although the rest of the included studies found beneficial modifications of the GM of newborns even when beginning intervention later [35,38]. These early interventions can restore the low *Bifidobacterium* presence in C-section infants within a week [31]. The intervention period is also important. In the present review, although some authors state that a minimum of 3 months would be needed to restore the GM of C-section infants by using probiotic strains [36], we have found that shorter intervention durations are also effective [28,35,37,39].

Regarding the administration of probiotics during pregnancy, the two studies included started the intervention in the last month of pregnancy. Whether these prenatal interventions exert an effect on C-section infants remains unclear. Mastromarino et al. [29] found that a multi-strain probiotic combination positively influenced the beneficial microbiota of breast milk, by means of as systemic effect exerted by the probiotics, but this effect was less evident in C-section newborns. On the contrary, Korpela et al. [36] observed that most of the cesarean-associated changes in the fecal microbiota of infants were corrected or reduced by a probiotic supplementation to mother and infant, indicating that that breastfeeding together with probiotic supplementation offered optimal results in terms of supporting the microbiota development in these infants. However, since both the mother and the infant received the same probiotic supplement, it was not possible to elucidate the role of the maternally ingested probiotic on the infant GM.

Another important point is the persistence of probiotic colonizers over time. In most of the studies in our review, the probiotic strain was found for either a short period of time or not at all in feces. Despite this, a beneficial effect of probiotic strains on the GM has been observed [33], demonstrating persistent changes in the infant microbial ecosystem after the end of the intervention in most of the studies. One explanation could be that probiotic strains help to create a microbial ecosystem that facilitates the growth of autochthonous beneficial bacteria, which would, in turn, be responsible for the health benefits observed. In this sense, it has been observed that intervention with prebiotics is able to increase *Bifidobacterium* populations by increasing the endogenous population of *Bifidobacterium* in healthy, term infants [44].

Regarding the use of these products by the general population, consumers and medical providers must bear in mind there are very important details that must always be available. These include the specific strain/s, the number of microorganisms, the treatment duration, the route of administration, the formulation, the shelf-life, and the storage conditions, which unfortunately are often missing. In this sense, it is worthy to mention that *Lactobacillus* nomenclature has recently been changed [45] based on several genetic approaches and markers, providing a better ecological and functional vision. As an example, following this new classification, *Lactobacillus reuteri* is now named *Lacticaseibacillus reuteri.* Consequently, the labels of probiotic products will need to be updated and scientists will need to take the new names into account for future publications and new patents. In addition, this will be critical when performing literature searches.

### 4.1. Strengths

With the exception of one study, all included studies were randomized controlled trials, giving the highest degree of evidence. The review includes recent studies and provides specific strains, doses, and intervention times. We have conducted the review based on PRISMA guidelines, selecting studies published since database inception. Moreover, most of the studies have low risk of bias.

### 4.2. Limitations

We found high variability regarding the strains and dosages of probiotic microorganisms alone or in combination with prebiotics, which makes it difficult to suggest a specific strain or dosage. In addition, the methodology regarding fecal collection and microbial analyses was heterogenous, which makes it difficult to compare results. In some studies, the influence of important factors such as feeding (breastfed or formula) and antibiotic intake were not taken into account.

## 5. Conclusions

The intake of probiotics, prebiotics, and synbiotics, especially during lactation, results in beneficial effects on the gut microbiota of newborns, especially C-section newborns. These interventions are more effective when ingestion begins soon after birth, especially for restoring the population of bifidobacteria. More interventional studies are needed to elucidate the optimal synbiotic combinations and the most effective strains and doses to achieve the optimal gut microbiota colonization of C-section newborns.

## Figures and Tables

**Table 1:** Characteristics of the studies selected.

Author/Year	Design	Population	Intervention	Control	Intervention Duration	Outcome
Yuniati, 2013 [28]	CT	*n* = 122 newborns *n* (IG) = 87 (50% CD) *n* (CG) = 81 (50% CD)	Mixed feeding plus *B. lactis* DSM 10140	Mixed feeding	From birth to 2 months	Increase of *B. lactis* in stool of IG compared to CG. In the intervention group, *B. lactis* was found in the 80% of the CD and in the 38% of the VD infants. Higher counts of Bifidobacteria in CD infants belonging to the IG compared to those in the CG at 1 month
Mastromarino, 2015 [29]	RCT-DB	*n* = 66 pairs pregnant female-newborns *n* (IG) = 33 (42.4% CD) *n* (CG) = 33 (31.3% CD)	Oral daily ingestion of 9 × 10^11^ of VSL# probiotic mixture: *Lactobacillus acidophilus* DSM 24735, *L. plantarum* DSM 24730, *L. paracasei* DSM 24733, *L. delbrueckii subsp. bulgaricus* DSM 24734, *Bifidobacterium* *longum* DSM 24736, *B. breve* DSM 24732, *B. infantis* DSM 24737, and *Streptococcus thermophilus* DSM 24731	Corn starch	From 36th week of pregnancy to 4 weeks after delivery	Beneficial gut microbiota instauration, especially in CD newborns. Significantly higher amounts of lactobacilli and bifidobacteria in colostrum and mature milk of probiotic treated women delivering vaginally, compared to CG
Baglatzi, 2016 [30]	RCT-DB	n = 198 CD newborns *n* (IG_1_) = 77 *n* (IG_2_) = 77 *n* (CG) = 44	Infant formula plus: IG_1_: 10^7^ CFU/g *B. lactis* CNCM I-3446 IG_2_: 10^4^ CFU/g *B. lactis* CNCM I-3446	Breastfeeding (min. 4 months)	From birth to 6 months of age	At 4 months, no differences were found regarding total bifidobacteria. In 85% of IG_1_ and 47% of IG_2_ feces, *B. lactis* was detected
Cooper, 2016 [31]	RCT-DB	n = 421 newborns n (IG) = 207 (44% CD) n (CG) = 214 (47% CD)	Infant formula plus 1 × 10^7^ CFU/g of *Bifidobacterium animalis* subsp. *lactis* CNCM I-3446 and 5.8 g/100 g of a mixture of bovine milk-derived oligosaccharides (BMOS)	Infant formula	From birth to 6 months of age	Infant formula supplemented with the synbiotic induced a bifidogenic effect in both delivering modes, but more explicitly correcting the low bifidobacterial level found in CD infants. Lowered fecal pH and improved fecal microbiota independently of the delivery mode
García-Ródenas, 2016 [32]	RCT-DB	*n* = 40 newborns *n* (IG) = 20 (50% CD) *n* (CG) = 20 (50% CD)	Infant formula plus 1.2 × 10^9^ CFU/L of *Lactobacillus reuteri* DSM 17938	Infant formula	From 72 hours after delivery until 6 months of age	Increase in *L. reuteri* in infants receiving the probiotic formula, independent of the delivery mode and age. *L reuteri* promoted the growth of other *Lactobacillus spp*. and strongly modulated the microbiota in CD babies
Bazanella, 2017 [33]	RCT-DB	*n* = 106 newborns *n* (IG) = 48 (42% CD) *n* (CG) = 49 (45% CD) *n* (RG) = 9 breastfed	Infant formula plus 10^7^ CFU/g of a mixture of *Bifidobacterium bifidum* BF3, *B. breve* BR3, *B. longum* BG7, *B. longum subspecies infantis* BT1	Infant formula	From delivery until 1 year of age	IG infants showed decreased occurrence of *Bacteroides* and *Blautia* spp. at month 1. No detectable long-term effects for gut microbiota assembly or function
Chien Chua, 2017 [34]	RCT-DB	*n* = 183 newborns *n* (IG_1_) = 52 CD *n* (IG_2_) = 51 CD *n* (CG) = 80 (38% CD)	Infant formula plus: IG_1_: 0.8 g/100 mL scGOS/Lcfos. IG_2_: 0.8 g/100 mL scGOS/Lcfos + *B. breve* M-16V (7.5 × 10^8^ CFU/100 mL)	Infant formula	From birth (1–3 days at the latest) until 16 weeks of age	Supplementation with both prebiotics (IG_1_) and synbiotics (IG_2_) in CD infants allows fast colonization from the first days of life, emulating the gut physiological conditions observed in vaginally delivered infants
Frese, 2017 [35]	RCT	*n* = 66 newborns *n* (IG) = 34 (32% CD) *n* (CG) = 32 (28% CD)	Breastfeeding plus a daily capsule containing 1.8 × 10^10^ CFU of *Bifidobacterium longum* subsp. *infantis* EVC001	Breastfeeding	From day 7 to day 28 of life	Increase in *Bifidobacteriaceae*, in particular *B. infantis*, in IG, persisting more than 30 days after probiotic supplementation ceased. Relative abundances of *Enterobacteriaceae, Clostridiaceae, Erysipelotrichaceae, Pasteurellaceae, Micrococcaceae*, and *Lachnospiraceae* diminished in IG compared to CG
Korpela, 2018 [36]	RCT-DB	*n* = 422 pairs pregnant female-newborns *n* (IG) = 199 (18% CD) *n* (CG) = 223 (20% CD)	Mothers: probiotic mixture containing 5 × 10^9^ CFU *Lactobacillus GG* (ATCC 53103), 5 × 10^9^ CFU *L. rhamnosus* LC705, 2 × 10^8^ CFU *Bifidobacterium breve* Bb99, and 2 × 10^9^ CFU *Propionibacterium freudenreichii ssp. shermanii* JS, twice a dayNewborns: same probiotic mixture as mothers, mixed with 0.8 g of GOS	Microcrystalline cellulose	Mothers: last month of pregnancy.Infants: from birth until 6 months of age	Daily *B. breve* and *L. rhamnosus* supplementation combined with breastfeeding is a safe and effective method to support the microbiota in CD and in antibiotic-treated infants
Hurkala, 2020 [37]	RCT	*n* = 148 C-section newborns *n* (IG) = 71 *n* (CG) = 77	Oral capsule containing 2 × 10^6^ CFU/day *Bifidobacterium breve* PB04 and *Lactobacillus rhamnosus* KL53A	Mother’s milk or formula	From delivery to 6 days of life	Supplementation of CD neonates with a mixture of *L. rhamnosus* and *B. breve* strains immediately after birth increases numbers of lactobacilli and bifidobacteria in their gut
Estorninos, 2021 [38]	RCT-DB	*n* = 226 newborns *n* (IG) = 114 (17% CD) *n* (CG) = 112 (18% CD) *n* (RG) = 70 breastfed (19% CD)	Infant formula containing 7.2 g/L bovine milk-derived oligosaccharides (MOS)	Infant formula	From 21–26 days of age until 6 months of life	Supplementation with MOS shifts the gut microbiota composition of CD infants towards that of vaginally delivered, breastfed infants
Phavichitr, 2021 [39]	RCT-DB	*n* = 290 C-section newborns *n* (IG_1_) = 81 *n* (IG_2_) = 82 *n* (CG) = 84 *n* (RG) = 43 breastfed	Infant formula containing: IG_1_: 0.8 g/100 mL scGOS/lcFOS and *B. breve* M-16v (1 × 10^4^ CFU/100 mL) IG_2_: 0.8 g/100 mL scGOS/lcFOS and *B. breve* M-16v (1 × 10^6^ CFU/100 mL)	Infant formula	From birth till 6 weeks of age	Both synbiotic formulas (IG_1_ and IG_2_) increased the bifidobacteria proportions and decreased the prevalence of *C. difficile.* Fecal pH was significantly lower while L-lactate concentrations and acetate proportions were significantly higher in both intervention groups compared to RG

CT: controlled trial; RCT: randomized controlled trial; DB: double-blind; *n*: sample size, IG: intervention group, CG: control group, RG: reference group; CD: cesarean delivery; GOS: galacto-oligosaccharides; scGOS: short chain galacto-oligosaccharides; lcFOS: long chain fructo-oligosaccharides.

## Data Availability

The publications analyzed for this systematic study can be accessed from their respective journals, whereby access restrictions may apply.

## Supplementary Materials

The following supporting information can be downloaded at: https://www.mdpi.com/article/10.3390/nu14020341/s1. Table S1. Search strategies used for this review, Table S2. Summary of assessing risk of bias according to ROB2 checklist.
