# Supplementary material for: The Impact of Probiotics, Prebiotics, and Synbiotics during Pregnancy or Lactation on the Intestinal Microbiota of Children Born by Cesarean Section: A Systematic Review
Source: Nutrients. 2022 Jan 14;14(2):341. doi: 10.3390/nu14020341 (PMC8778982; doi:10.3390/nu14020341)
Supplement: Supplementary file 1 [file nutrients-14-00341-s001.zip › nutrients-1531697-supplementary.pdf]

SUPLEMMENTARY MATERIAL

Table S1. Search strategies used for this review.

**Search Strategy**	**Articles**
**Pubmed**<br><br>("probiotic*"[Title/Abstract] OR "symbiotic"[Title/Abstract] OR "prebiotic"[Title/Abstract] OR "Lactobacil*"[Title/Abstract] OR "bifidobacteri*"[Title/Abstract] OR "oligosaccharid*"[Title/Abstract] OR "fructo*"[Title/Abstract] OR "galacto*"[Title/Abstract]) AND ("pregnan*"[Title/Abstract] OR "gestation"[Title/Abstract] OR "lactation"[Title/Abstract] OR "breastfeeding"[Title/Abstract] OR "formula"[Title/Abstract]) AND ("cesarean"[Title/Abstract] OR "caesarean"[Title/Abstract] OR "c-section"[Title/Abstract]) AND ("trial"[Title/Abstract]) NOT ("mice"[Title/Abstract] OR "mouse" [Title/Abstract] OR "rat"[Title/Abstract] OR "vitro"[Title/Abstract] OR "pig"[Title/Abstract] OR "monkey"[Title/Abstract]) NOT ("review"[Title/Abstract])	19
**Scopus**<br><br>TITLE-ABS-KEY (probiotic* OR prebiotic* OR symbiotic* OR Lactobacill* OR Bifidobacteri* OR oligosaccharide* OR fructo* OR galacto*) AND TITLE-ABS-KEY (mother OR pregnan* OR gestation OR lactation OR breastfeeding OR formula) AND TITLE-ABS-KEY (cesarean OR caesarean OR c-section) AND TITLE-ABS (trial) AND NOT TITLE-ABS-KEY (mice OR mouse OR rats OR vitro OR pig* OR monkey) AND (LIMIT-TO (DOCTYPE,"ar")) AND (LIMIT-TO (LANGUAGE,"English"))	38
**Web of Science**<br><br>AB=(probiotic* OR Lactobacill* OR Bifidobacteri* OR synbiotic* OR prebiotic* OR oligosaccharide OR fructo* OR galacto*) AND AB=(mother OR pregnan* OR lactation OR breastfeeding OR formula OR gestation) AND AB=(cesarean OR caesarean OR c-section) AND (AB=(trial) OR TI=(trial)) NOT AB=(mice OR mouse OR rats OR vitro OR pig* OR monkey)	18

ABS, AB: Abstract; TS: Topic; TI: Title

Table S2. Summary of assessing risk of bias according to ROB2 checklist

Author, year [Ref]	Selection bias		Performance bias	Detection bias	Attrition bias	Reporting bias	Overall Risk of Bias
	Random sequence generation	Allocation concealment					
**Yunati, 2013[28]**	High	Unclear	Unclear	Low	Low	Low	**High**
**Mastromarino, 2015[29]**	Low	Low	Low	Low	Low	Low	**Low**
**Baglatzi, 2016[30]**	Low	Low	Low	Low	Low	Low	**Low**
**Cooper, 2016[31]**	Unclear	Low	Low	Low	Low	Low	**Unclear**
**García-Ródenas, 2016[32]**	Unclear	Low	Low	Low	Low	Low	**Low**
**Bazanella, 2017[33]**	Low	Low	Low	Low	Low	Low	**Low**
**Chien-Chua, 2017[34]**	Unclear	Low	Low	Low	Low	Low	**Unclear**
**Frese, 2017[35]**	High	Unclear	Low	Low	Low	Low	**High**
**Korpela, 2018[36]**	Low	Low	Low	Low	Low	Low	**Low**
**Hurkala, 2020[37]**	Low	Low	Unclear	Low	Low	Low	**Low**
**Estorninos, 2021[38]**	Low	Low	Low	Low	Low	Low	**Low**
**Phavichitr, 2021[39]**	Unclear	Low	Low	Low	Low	Low	**Unclear**

Considerations:
Selection bias (including Random sequence generation & Allocation concealment)
Performance bias (Blinding of participants and personnel)
Detection bias (Blinding of outcome assessment)
Attrition bias (Incomplete outcome data)
Reporting bias (Selective reporting)
Overall Risk of bias: Low (for all domains), Unclear (in at least one domain), High (in at least one domain/ or unclear for multiple domains).
